# Spontaneous cerebrospinal fluid rhinorrhoea and aspiration pneumonitis following initiation of continuous positive airway pressure treatment for obstructive sleep apnoea

**DOI:** 10.1002/rcr2.435

**Published:** 2019-05-20

**Authors:** Sanjiwika Lalanjani Wasgewatta, Nathan Manning, Michael Redmond, Diane Howard, Subash Shanthakumar Heraganahally

**Affiliations:** ^1^ Department of Respiratory and Sleep Medicine Royal Darwin Hospital Darwin Northern Territory Australia; ^2^ Department of Medical Imaging Royal Darwin Hospital Darwin Northern Territory Australia; ^3^ Department of Medical Imaging, Florey Institute of Neuroscience Melbourne Victoria Australia; ^4^ Department of General Surgery/Neurosurgery Royal Darwin Hospital Darwin Northern Territory Australia; ^5^ Department of General Medicine Royal Darwin Hospital Darwin Northern Territory Australia; ^6^ College of Medicine and Public Health, Flinders University Adelaide South Australia Australia; ^7^ Northern Territory Medical School Charles Darwin University Darwin Northern Territory Australia

**Keywords:** Aspiration pneumonitis, cerebrospinal fluid, continuous positive airway pressure, intracranial hypertension, obstructive sleep apnoea

## Abstract

Continuous positive airway pressure (CPAP) therapy is very often the treatment of choice for obstructive sleep apnoea (OSA). The association between intracranial hypertension and spontaneous cerebrospinal fluid (CSF) rhinorrhoea is being increasingly recognized among patients with OSA. However, spontaneous CSF rhinorrhoea following initiation of CPAP therapy for OSA is very rarely documented in the literature. In this report, we describe a 53‐year‐old woman with severe OSA who, while being evaluated for possible intracranial hypertension, developed spontaneous CSF rhinorrhoea and CSF aspiration pneumonitis as a complication of CPAP therapy. Magnetic resonance imaging confirmed fluid tracks at the skull base, and a nasal swab demonstrated positive β2‐transferrin. Computer tomography (CT) chest showed findings consistent with CSF aspiration pneumonitis. Resolution of both CSF leak and pneumonitis were noted following treatment with azetozolamide and curative endoscopic trans‐nasal surgery along with ventriculoperitoneal shunt.

## Introduction

Continuous positive airway pressure (CPAP) therapy is very often the treatment of choice for moderate to severe obstructive sleep apnoea (OSA). The association between intracranial hypertension and spontaneous cerebrospinal fluid (CSF) rhinorrhoea is being increasingly recognized among patients with OSA 1, 2, 3, 4. However, spontaneous CSF rhinorrhoea following initiation of CPAP therapy for OSA is very infrequently documented in the literature 5, 6, 7. In this report, we describe a 53‐year‐old woman with severe OSA who, while being investigated for benign intracranial hypertension, developed spontaneous CSF rhinorrhoea and CSF aspiration pneumonitis following initiation of CPAP therapy.

## Case Report

A 53‐year‐old woman presented to the emergency department with a 4‐week history of rhinorrhoea and post‐nasal drip, exacerbated by coughing and bending forward. Onset of rhinorrhoea was noted three days following the initiation of nasal CPAP therapy for severe OSA (apnoea hypopnea index (AHI) of 35/h, more severe during Rapid eye movement sleep, AHI 82/h). CPAP therapy was initiated at a pressure of 11 cm H_2_O following a CPAP titration study.

The patient's past medical history included poorly controlled type 2 diabetes and hypertension. The patient was also undergoing investigation for constant headaches for several months to years, and benign intracranial hypertension was being considered in the differential diagnosis. She also reported recent onset of dry cough during this presentation, which coincided with the onset of rhinorrhoea. There was no other significant past medical history; in particular, she did not report previous cranial or sinus trauma or cranio‐facial surgery. Clinical examination showed that she was febrile at presentation, and other vital signs were unremarkable, including oxygen saturation of 97% on room air. Fundoscopy demonstrated bilateral papilloedema. Respiratory examination showed clear breath sounds, with no crackles or signs of consolidation or pleural effusion. Systemic and neurological examinations were unremarkable; in particular, there were no signs of meningitis.

Lumbar puncture showed an opening CSF pressure of 24 cm H_2_O. The CSF fluid cell count, glucose and proteins were within normal range. A blood test showed raised inflammatory markers with a C‐reactive protein (CRP) of 122 mg/L and raised white cell count of 22 × 109/L with neutrophilic predominance. Liver function test was normal. Connective tissue disease, vasculitis screening, and blood cultures were negative. Derangement of renal function and proteinuria were noted and were considered to be secondary to poorly controlled type 2 diabetes.

**Figure 1:**
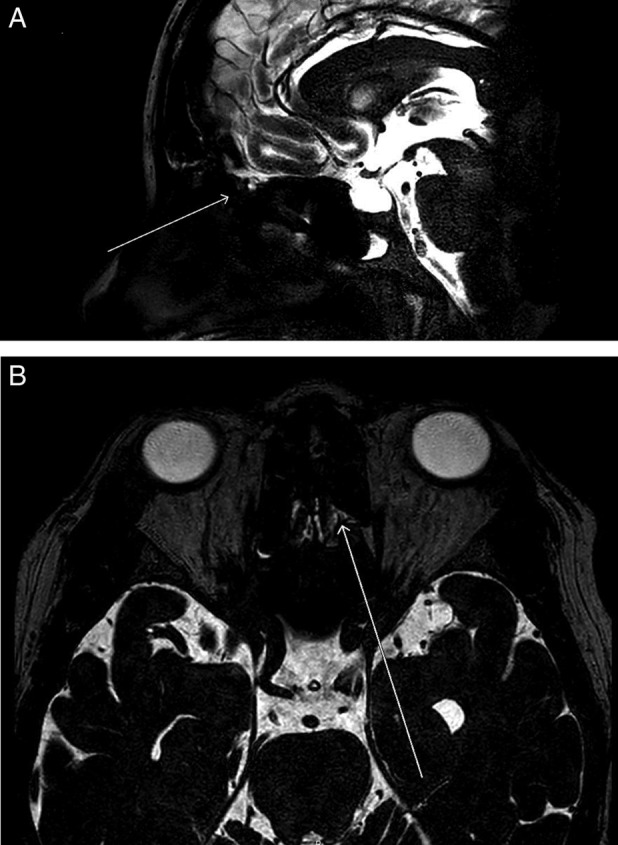
(A, B) Sagittal T2‐weighted magnetic resonance imaging scan demonstrating CSF leak from the anterior cranial fossa via the cribriform plate to the superior nasal cavity.

**Figure 2:**
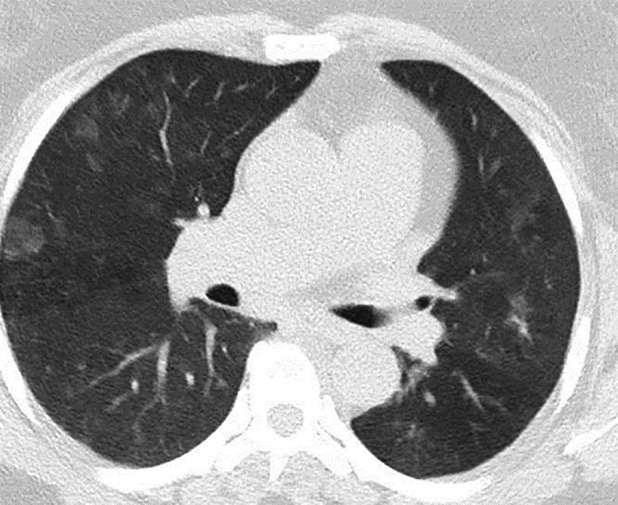
Bilateral, predominantly basal, ground‐glass opacities noted on computer tomography chest suggestive of CSF aspiration pneumonitis.

Rhinorrhoea was confirmed to be secondary to CSF leak by nasal swab, demonstrating positivity for β2‐transferrin. Magnetic resonance imaging (MRI) of the head demonstrated an “empty” sella turcica, enlargement of Meckel's cave bilaterally, and bilateral optic nerve sheath effusions, along with flattening of the posterior optic disc consistent with intracranial hypertension. There was also cortical thinning of the floor of the sella and cribriform plate. Fluid was also demonstrated within the frontal and sphenoid sinuses. A computer tomography venogram demonstrated absent left transverse sinus and sigmoid sinus, and the venous drainage was noted predominantly through right transverse sinus and sigmoid sinus. Dedicated T2 MRI scanning through the anterior cranial fossa, skull base, and paranasal sinuses was performed to identify the site of the CSF leak, which demonstrated multiple fluid tracks noted in the region of the cribriform plate (Fig. 1A, B). CT scan of the chest showed bilateral, predominantly basal, ground‐glass opacities, which were considered to be secondary to CSF aspiration pneumonitis (Fig. 2).

The patient reported significant improvement in headache and rhinorrhoea following initiation of azetozolamide. Subsequently, the skull base was repaired via an endoscopic trans‐nasal surgery, and a ventriculoperitoneal shunt was placed for CSF diversion. During the follow‐up visits, she had no further headaches and was free of CSF leak. Her pulmonary symptoms improved spontaneously, and follow‐up chest CT showed complete resolution of the previously noted opacities within one week of resolution of CSF rhinorrhoea.

## Discussion

The case presented here is a very rare and uncommonly reported complication of CPAP treatment for OSA (CSF rhinorrhoea and CSF aspiration pneumonitis). Previous studies have documented an association between OSA and spontaneous cerebrospinal fluid rhinorrhoea (SCSFR), and with a diagnosis of OSA, the odds of SCSFR is noted to be around 4.73 times higher than the control cohorts 2. Similarly, other studies have shown that patients undergoing surgery for SCSFR repair are noted to have OSA (14.8%) and hypertension (85.7%) 3. Furthermore, in patients undergoing endoscopic surgical repair for CSF rhinorrhoea, patients with SCSFR are more likely than their non‐spontaneous counterparts to have a diagnosis of OSA (30.0 vs. 14.3%), and it is more common in female than male patients (84.3 vs. 41.1%) 8.

Repeated apnoea and hypoxia are thought to increase the intracranial pressure in patients with OSA 5. However, there are only a few reported cases of spontaneous CSF rhinorrhoea following initiation of CPAP therapy without prior craniofacial trauma or surgery in the literature 5, 6, 7. The mechanism of CSF leak following initiation of CPAP therapy is thought to be related to alterations in the intracranial pressure and CSF venous pressure, leading to changes in the transdural pressure gradient 5. Furthermore, the presence of a pre‐existing congenital abnormality at the cribriform plate, trauma, or prior surgical intervention to cranio‐facial structures may increase the risk 7, 9. In our patient, it is likely that, in the presence of chronic intracranial hypertension and cortical thinning of the floor of the sella and cribriform plate, initiation of CPAP might have led to CSF rhinorrhoea.

Although rare, it is noted that the most frequently reported complications of CSF rhinorrhoea are meningitis and pneumocephalus 6, 7, 9. Aspiration pneumonitis secondary to CSF rhinorrhoea is infrequently reported or recognized 10, 11. It is more than likely that this condition is under‐reported or under‐recognized in clinical practice. Patients with CSF, aspiration pneumonitis may present with signs and symptoms suggestive of recurrent pneumonitis/bronchiolitis or can mimic bacterial infection and may only partially respond to anti‐microbial therapy. Patients may report previous cranial or sinus trauma or cranio‐facial surgery. Laboratory investigations may demonstrate raised inflammatory markers, such as high white cell count and raised CRP, as in our patient. Radiology may demonstrate waxing and waning pulmonary infiltrates, especially in patients with chronic intermittent CSF fluid aspiration. Pulmonary function test (PFT) and bronchoscopy are likely to be normal in the acute setting. However, if the CSF aspiration pneumonitis is intermittent and chronic, PFT may demonstrate a mixed obstructive—restrictive pattern. Lung biopsy may demonstrate features of bronchiolitis obliterans. Delay in the diagnosis of CSF aspiration pneumonitis can occur. However, demonstration of β‐2‐transferrin and high glucose level in the nasal secretion will facilitate early diagnosis in suspected cases 10, 11.

We present this case to add to the limited number of reported cases. We also believe that this report may encourage other clinicians to report similar cases to better understand the mechanism, prevention, and early diagnosis and management of this condition. Nevertheless, a history of headaches and visual symptoms may need to be considered in patients diagnosed to have OSA prior to commencing CPAP therapy. If there is a history of intracranial hypertension or clinical symptoms of intra cranial hypertension or previous cranial or sinus trauma or cranio‐facial surgery, careful monitoring for any evidence of CSF leak following the commencement of CPAP therapy and CSF aspiration pneumonitis needs to be considered in the relevant clinical context. Accurate diagnosis and treatment with surgical intervention can be curative 11. Furthermore, oral mask interface may need to be considered in the management of OSA in patients who have clinical symptoms suggestive of intracranial hypertension.

### Disclosure Statement

Appropriate written informed consent was obtained for publication of this case report and accompanying images.
